# An Emptying Quiver: Antimicrobial Drugs and Resistance

**DOI:** 10.3201/eid1106.050471

**Published:** 2005-06

**Authors:** J. Todd Weber, Patrice Courvalin

**Affiliations:** * Centers for Disease Control and Prevention, Atlanta, Georgia, USA;; † Institut Pasteur, Paris, France

**Keywords:** Antimicrobial, Antibiotic, Resistance

**Figure 1:**
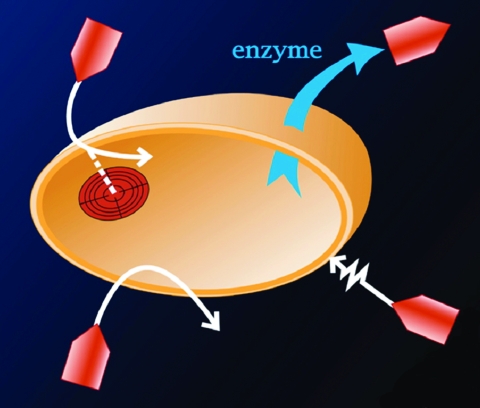
Schematic representation of mechanisms of resistance to antimicrobial agents.

Since the dawn of the antibiotic era, resistance has shadowed the success of infectious disease therapy. In his 1945 Nobel Prize acceptance speech, Alexander Fleming noted the danger of resistance: "It is not difficult to make microbes resistant to penicillin in the laboratory by exposing them to concentrations not sufficient to kill them, and the same thing has occasionally happened in the body…. Moral: If you use penicillin, use enough" (1). Sixty years later, our understanding of resistance has grown vastly more sophisticated and the proliferation of new antimicrobial drugs has engendered an equally varied collection of resistance mechanisms (Figure 1). Resistance is now an important problem in virtually all areas of infectious diseases, including viral, bacterial, fungal, and parasitic diseases.

In a 2003 Institute of Medicine report, Microbial Threats to Health, antimicrobial resistance was noted as a paramount microbial threat of the twenty-first century (2). Some strains of pathogenic bacteria are now resistant to essentially all available antimicrobial drugs, and some remain susceptible to only one. At the same time, what once was an apparent deluge of antimicrobial drug development is now barely a trickle. The lack of new drug classes is a consequence of difficulties in discovery of new compounds that has persisted for many years. In addition, pharmaceutical companies are finding in industrialized nations more potent markets for other disease treatments and lower profit in nonindustrialized countries (3,4). This trend is reflected in the absence of any novel class of antibacterial drug approved for use in the United States between 1968 and 2000. Indeed, most of the new drugs approved since 1968 have been chemical modifications of existing drugs. However, since 2000, two new drug classes have been approved by the U.S. Food and Drug Administration (5,6). Whether this trend will continue is unclear and does not obviate the need for more new classes.

Barring the arrival in the near future of new antimicrobial drugs that are effective against disparate organisms, we are left with imperfect tools to control drug resistance. With a notable exception, vaccines have not been produced that address the problem of resistance (7). Infection control in healthcare settings, which is essential for preventing transmission of susceptible and resistant microorganisms alike, remains imperfect. Reducing the discretionary use of antimicrobial drugs when possible is helpful, but even if we use these drugs with exquisite precision, resistance will continue to evolve and spread. Ensuring adherence to multidrug regimens to prevent emergence of resistance requires uninterrupted drug supplies and is vulnerable to human inconstancy. Finally, efforts to modify behavior (sexual and otherwise), will always have limitations in a free society. All of these shortcomings emphasize the critical role of research and dissemination of information. For that reason, this issue of Emerging Infectious Diseases is devoted to antimicrobial resistance and highlights both burgeoning and neglected areas.

Articles address antimicrobial resistance in pathogens from the community, healthcare settings, and agriculture, among children and adults, and in several countries. In the case of community-associated methicillin-resistant *Staphylococcus aureus* (MRSA), articles cover outbreaks in Uruguay and in a US hospital nursery and maternity unit, emergence of a particular clone in Canada, prevalence in US emergency department patients, characteristics of patients admitted to a Swiss hospital, and the severity of this infection in pediatric patients. One article estimates hospitalizations associated with MRSA infection. These articles show some of the changing spectrum of disease and populations affected by this pathogen. The success of a relatively new vaccine against resistant *Streptococcus pneumoniae* and its impact on resistant infections is described. Resistance towards macrolides and structurally related drugs is the subject of several articles. Antimicrobial-resistant foodborne infections are addressed in articles on *Salmonella* spp. and *Campylobacter jejuni.* Several articles discuss resistant organisms that are largely problems in healthcare settings or among persons with underlying illness, such as extended-spectrum β-lactamase (ESBL) producing *Escherichia coli*, vancomycin-resistant *Enterococcus faecium*, and gram-negative bacilli. Multidrug-resistant tuberculosis, perennially transmitted inside and outside healthcare and other institutional settings, is also discussed in 2 articles. An article on *Trypanosoma brucei gambiense* describes the importance and difficulty in determining resistance in parasitic infections, which can have countrywide implications for treatment, control, and use of resources.

This issue does not cover resistance in malaria, gonorrhea, and HIV infection. An estimated 300–500 million clinical cases of malaria occur each year, and resistance exists to some extent to nearly all available antimalarial drugs. Efforts are under way to produce new drugs, but one of the most efficacious candidates has run into economic and manufacturing obstacles (8,9). Gonorrhea treatment and control are becoming increasingly difficult because of increases in resistance to multiple classes of drugs and the discontinued production of a preferred oral medication (10,11). Antiretroviral drug resistance has been well demonstrated in countries in which the standard of care mandates treatment of HIV-infected persons. The imminent widespread use of antiretroviral drugs in countries with large populations infected with HIV, but for whom treatment has not been provided, will carry with it the specter of global resistance, thereby threatening the intended benefits (12). Finally, in the absence of vaccine, the long-anticipated arrival of a global influenza pandemic, caused by H5N1 or another strain, would have potentially devastating consequences with respect to resistance. The potential for antiviral drug resistance to develop during treatment is manifest. The likely increased use of oral antibacterial drugs for patients with possible influenza and for prophylaxis and treatment of secondary bacterial pneumonia would enhance the existing evolutionary pressure toward resistance. The predictably large numbers of secondary bacterial pneumonias may be caused by established resistant pathogens (e.g., pneumococcus) and emerging ones (e.g., community-associated MRSA).

Fleming's warning about inappropriate use has resonance today. Several articles describe the importance of the appropriate use of antimicrobial drugs as well as the difficultly of enforcement. The hope of preserving the effectiveness of existing drugs through appropriate use as well as the urgent need for the development of new drugs are both represented by the artwork featured on the cover of this issue. We hope to promote greater awareness among our readers of the strong link between antimicrobial drug use and the development of resistance and to make clear that improving use in community, healthcare, and agriculture settings, combined with other strategies, is imperative if we are to confront effectively the further development and spread of antimicrobial resistance.

**Figure 2:**
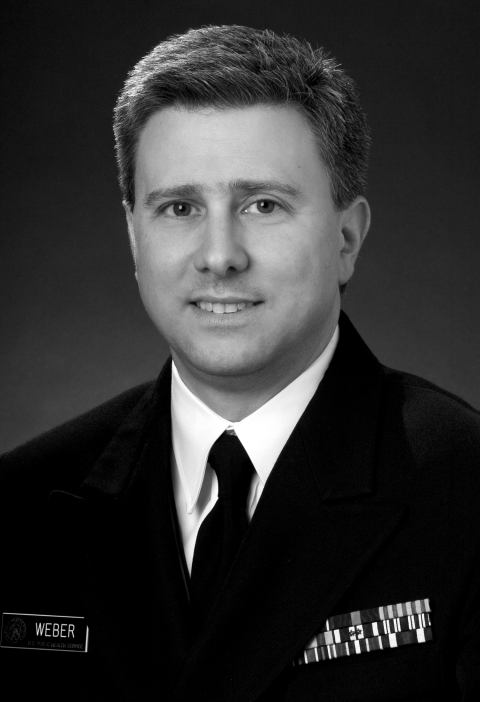
J. Todd Weber Dr. Weber is the director of the Office of Antimicrobial Resistance, National Center for Infectious Diseases, Centers for Disease Control and Prevention. He is responsible for coordinating antimicrobial resistance activities at CDC and co-chairs the federal Interagency Task Force on Antimicrobial Resistance. He works with other agencies, state governments, medical societies, and other public and private organizations to enhance antimicrobial resistance prevention and control, surveillance and response, applied research, and training.

**Figure 3:**
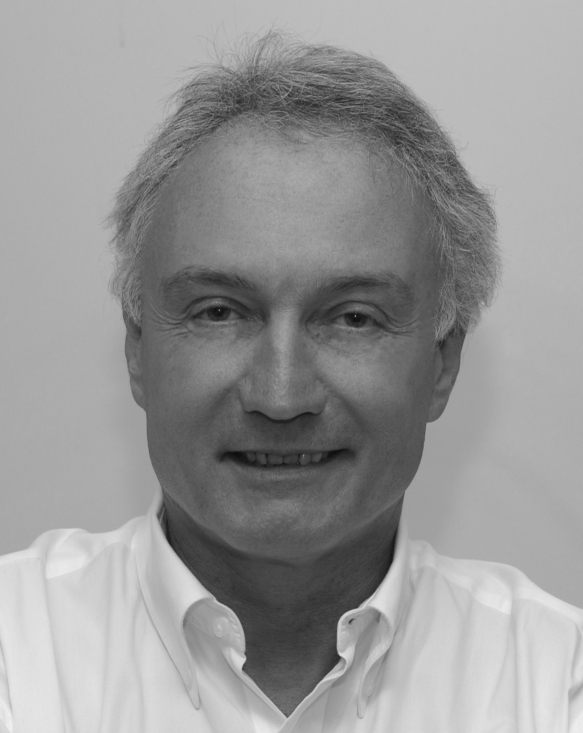
Patrice Courvalin - Dr. Courvalin is professor and head of the Antibacterial Agents Unit, National Reference Center for Antibiotics, Institut Pasteur, Paris. He is a member of multiple committees and professional organizations in Europe and around the world and a prolific author on infectious diseases and antimicrobial resistance topics. He serves on the editorial board of several international journals, including Emerging Infectious Diseases.
